# The role of research in improving responsiveness of palliative care to migrants and other underserved populations in the Netherlands: a qualitative interview study

**DOI:** 10.1186/s12904-020-00701-2

**Published:** 2021-01-06

**Authors:** M. Torensma, B. D. Onwuteaka-Philipsen, X. de Voogd, D. L. Willems, J. L. Suurmond

**Affiliations:** 1 Department of Public and Occupational Health, Amsterdam UMC Expertise center for Palliative Care and Amsterdam Public Health Research Institute, Amsterdam UMC, University of Amsterdam, Meibergdreef 9, Amsterdam, Netherlands; 2 Department of Public and Occupational Health, Amsterdam UMC Expertise center for Palliative Care and Amsterdam Public Health Research Institute, Amsterdam UMC, Vrije Universiteit Amsterdam, de Boelelaan, 1117 Amsterdam, Netherlands; 3 Department of General Practice, Amsterdam UMC Expertise center for Palliative Care and Amsterdam Public Health Research Institute, Amsterdam UMC, University of Amsterdam, Meibergdreef 9, Amsterdam, Netherlands

**Keywords:** Palliative care, Research, Responsiveness, Underrepresented populations, Migrant patients

## Abstract

**Background:**

The aging of migrant populations across Europe challenges researchers in palliative care to produce knowledge that can be used to respond to the needs of the growing group of patients with a migration background and address ethnic disparities in palliative care. The aim of this study was to identify what factors influence researchers’ efforts to address responsiveness of palliative care to patients with a migration background and other underserved populations in their projects.

**Methods:**

We conducted semi-structured interviews with 11 researchers involved in seven projects under the Dutch national program for palliative care innovation.

**Results:**

Researchers’ efforts to address responsiveness of palliative care in their projects were influenced by individual factors, i.e. awareness of the need for responsiveness to patients with a migration background; experience with responsiveness; and, differences in perceptions on responsiveness in palliative care. Researchers’ efforts were furthermore influenced by institutional factors, i.e. the interaction with healthcare institutions and healthcare professionals as they rely on their ability to identify the palliative patient with a migration background, address the topic of palliative care, and enrol these patients in research; scientific standards that limit the flexibility needed for responsive research; and, the responsiveness requirements set by funding agencies.

**Conclusion:**

Researchers play a key role in ensuring research addresses responsiveness to patients with a migration background. Such responsiveness may also benefit other underserved populations. However, at times researchers lack the knowledge and experience needed for responsive research. To address this we recommend training in responsiveness for researchers in the field of palliative care. We also recommend training for healthcare professionals involved in research projects to increase enrolment of patients with a migration background and other underrepresented populations. Lastly, we encourage researchers as well as research institutions and funding agencies to allow flexibility in research practices and set a standard for responsive research practice.

**Supplementary Information:**

The online version contains supplementary material available at 10.1186/s12904-020-00701-2.

## Background

The aging of migrant populations across Europe challenges researchers in palliative care to produce knowledge that can be used to respond to the needs of the growing group of patients with a migration background and address ethnic disparities in palliative care [1–4]. The term migration background is used in the Netherlands to refer to both migrants and their descendants. According to the definition by Statistics Netherlands, someone has a migration background if that person or at least one of their parents was born outside the Netherlands [5]. In the Netherlands, older persons with a non-western migration background are expected to make up 17% of the population of adults aged 65 and older in 2060 [6]. Patients with an ethnic minority or migration background are, on average, found to have lower awareness and utilization of palliative care services [3], experience lower rates of referrals to palliative care services [7], and experience communication difficulties and dissatisfaction [3, 8].

In theory, palliative care research can contribute to diminishing ethnic disparities; it can help to identify and understand determinants of disparities, identify and evaluate interventions to eliminate them and contribute to quality improvement and innovation of palliative care [4, 9]. An understanding of the role of culture in end-of-life care, for example, has previously been identified as a priority for research [10]. Efforts to reduce disparities through research, however, depend on the ability and commitment of researchers to recruit study participants amongst migrant and other underserved populations.

Recruiting study participants in palliative care research is challenging, in general, as involving terminally ill patients raises ethical as well as methodological issues [11]. E.g. it is difficult to identify ‘the’ palliative patient, to address gatekeeping by healthcare professionals concerned about burdening their patients, and to ensure informed decision-making and informed consent. The latter is a challenge in particular because patients often associate palliative care with terminal care and researchers search for ways to diminish the emotional impact of patient information material [11].

Recruiting study participants with an ethnic minority or migration background in palliative care research may be additionally challenging. There is a lack of standardized collecting and reporting of patients’ ethnicity and migration background, patients’ preference for non-disclosure and curative treatment further complicate informed decision-making in palliative care research, and research participation rates are generally low [12–14].

Low research participation rates among patients with an ethnic minority or migration background occur across all types of health related research. They exist, in part, because these patients experience barriers to participation. Reported barriers include mistrust in research or researchers; low awareness of and ineffective communication about research opportunities; lack of education about research participation; and lack of cultural competence by research teams [12, 15]. Although not many studies have looked into palliative care research specifically, similar barriers to participation seem to exist [16]. It appears that current research practice is not responsive to patients with an ethnic minority or migration background, i.e. it does not adequately respond to their need for information or differing cultural perspective. The consequent underrepresentation of patients with an ethnic minority or migration background undermines research’ ability to contribute to responsiveness of palliative care and diminish disparities.

Several strategies exist to ensure health care organizations are responsive to patient diversity [17]. These strategies, such as the standards for equity in healthcare for migrants and other vulnerable groups, help increase accessibility and quality of services for users who differ from assumed norms [18]. Patient groups underserved due to attributes other than ethnic minority or migration background, e.g. socioeconomic status, geographical location, gender, religion, age, sexual orientation or disability, are also intended to benefit from responsiveness.

Measures that contribute to responsiveness of research have also been identified [12, 15, 19]. As these require efforts in all stages of a research project, lead researchers play a key role in establishing responsiveness. In the Netherlands, the national program for palliative care innovation (*Palliantie. Meer dan Zorg*), funded by The Netherlands Organization for Health Research and Development, brought about a considerable number of research, intervention and education projects that aim to improve palliative care. In this study we aimed to identify what factors influence researchers’ effort to address responsiveness of palliative care to patients with a migration background in their projects.

## Methods

### Design

We conducted semi-structured interviews with researchers involved in projects under the national program for palliative care innovation. This study was part of a project that aimed to develop a self-assessment instrument to help researchers assess and find ways to improve their projects’ responsiveness to diversity, in light of the aging migrant population [20]. The data gathering for this study had the combined purpose of answering our research questions, as well as informing the development of the self-assessment instrument. Due to the latter we conducted interviews at two time points, i.e. fall 2017 and spring 2018 (see Table 1. for further specification of time points and respondents).

**Table 1:** Description of research projects and interview respondents

		T1			T2		
Project	General aim	#Respondents	Respondent ID	Remarks	#Respondents	Respondent ID	Remarks
1	Evaluate and strengthen a procedure to improve palliative homecare by improving cooperation between GPs and community nurses	1	2017.09	Same researcher interviewed about project 1 & 2	1	2018.09	
2	Assess the palliative care needs and barriers to care for homeless people	1	2017.10	Same researcher interviewed about project 1 & 2	1	2018.10	
3	Develop, implement and evaluate a palliative care trajectory between different care settings	1	2017.11		2	2018.11	
4	To study goals set by patients with cancer and their physicians before starting systemic treatment and assess whether these goals are met	1	2017.12	Same researcher interviewed T1 & T2	1	2018.12	Same researcher interviewed T1 & T2
5	Develop, implement and evaluate an advance care planning intervention for primary care practice	1	2017.13	Same researcher interviewed T1 & T2	1	2018.13	Same researcher interviewed T1 & T2
6	Strengthen shared decision-making at the end of life by developing and implementing methods to engage all stakeholders in shared-decision making in palliative care				1	2018.14	No interview at T1^a^
7	Strengthen shared decision-making at the end of life shared by developing and implementing decision-making tools				2	2018.15	No interview at T1^a^

^a^These projects were purposively selected to ensure diversity in status of implementation; they were yet to start at T2 and could offer distinct insights from that stage

### Sample

We interviewed 11 researchers of diverse scientific- and methodological background, i.e. quantitative or qualitative, and with varying years of experience working as a researcher. The researchers were involved in seven projects under the national program for palliative care innovation. The projects aimed to implement and evaluate interventions and innovate palliative care (see Table 1.). We selected projects through one of the palliative care consortia in the Netherlands; a partnership between regional palliative care networks representing palliative care providers, IKNL (Quality institute for oncological and palliative research and practice) and two centres of expertise on palliative care. The projects were purposively selected based on diversity in focus and status of implementation to ensure data saturation. We were acquainted with most researchers through the consortium. Researchers were invited for an interview via email. None refused to participate. There was no compensation for participation. Interviews were face-to-face and took place in the workplace of the interviewed researcher, while no one else was present.

### Data collection & analysis

MT conducted all interviews. Interviews were conducted in Dutch. We used a semi-structured interview guide which included questions on researchers’ perception of diversity, responsiveness and their role in contributing to responsiveness of palliative care in their projects. It also included suggestions for strategies to improve responsiveness roughly following the domains of the equity standards: equity in policy; equitable access and utilisation; equitable quality of care; equity in participation; and, promoting equity [18]. The interview guide is included as an attachment (see Supplementary file 1). In the interviews we discussed the feasibility of implementing these strategies to identify factors influencing researchers’ effort to contribute to responsiveness of palliative care in their projects. Interviews on average took 50 min. All interviews were recorded and transcribed verbatim. The data was managed using MaxQDA.

We analysed the transcripts of the interviews using thematic analysis [21]. Authors MT and XdV reviewed the data to identify key themes and subthemes. A coding system was developed by coding two interviews individually and comparing and adjusting codes. MT subsequently coded all transcripts according to this coding system. Codes and corresponding quotes were discussed among MT, JS and BOP. During this process codes were refined by adding, subtracting, combining or splitting codes resulting in the final thematic framework. E.g. codes organized by impeding and facilitating factors in the different stages of a research project were later structured according to individual level and institutional level factors. The final results were discussed with MT, XdV, JS, BOP and DW to provide answers to the research question.

## Results

Various factors influenced researchers’ efforts to address responsiveness of palliative care in their projects. These factors either enabled or prevented researchers from contributing to improved responsiveness. We found factors at play on the individual level, i.e. researchers’ awareness, perception and experience, as well as factors at play on a more institutional level, i.e. in the interaction with healthcare professionals, healthcare institutions, research institutions and funding agencies.

When asked about diversity and responsiveness researchers oftentimes also spoke about underserved populations other than patients with a migration background, e.g. patient groups with a low socioeconomic status, low health literacy or palliative patients with a non-cancer diagnosis or comorbidity.

### Individual level

#### Awareness

To contribute to responsiveness of palliative care in their projects, researchers first had to acknowledge a need for responsiveness by recognizing that patients with a migration background are underserved in palliative care as well as underrepresented in research. As the palliative care innovations researchers were implementing in their projects were ‘new terrain’, some researchers did not anticipate a need for responsiveness (Quote #01).

***#01***″ *We started with the intervention and the people you reach, in fact, are the people who are very interested in palliative care. Who say this is what we need, it’s a great way to work.*. *.. And I have no idea if we reached healthcare professionals with a Dutch or western migration background more easily than healthcare professionals with a non-western migration background. That we unconsciously introduce something that is not at all appealing to people with a non-western migration background. I just don’t know, you see?” (Researcher 2018.09)*

In reaction to the question why patients with a migration background had not been taken into account researchers often responded it just had not been on their mind. After an increase in awareness, e.g. between interview time points, however, researchers expressed they more actively considered patient diversity (quote #02). Awareness of a need for responsiveness to patient diversity increased most effectively when the issue was raised by palliative care practice (quote #03).

***#02*** *“I am currently writing a project on bereavement care. And the fact that I am becoming more aware of diversity in my current project, makes me consider it more in the bereavement care project. And sometimes I tell colleagues they have to consider it too.” (Researcher 2017.13)*

***#03*** *“Yes, because … the current tool, the booklet comprises quite a large number of pages. When we were at the ... [Hospital with a predominantly low SES population] they said ‘gee, can you summarize this on one A4? Because our patients won’t be able to use this at all.’ Well, and then of course I couldn’t and I was quite convinced of the quality of our tool also. But at the moment I do understand that it could, and should, be a bit more simple.” (Researcher 2018.15)*

#### Perceptions

Researchers’ perception of responsiveness – how they understood responsiveness and the efforts it requires – differed. During the interviews several researchers iterated the aim of their research project is to improve care for *all* patients. They referred to the concept of patient centred care, where responsiveness is realised in the interaction between professional and patient. Researchers mentioned that in palliative care in particular there is room for a patient centred approach, as it looks individually at each patient’s needs for quality of life (quote #04). They pointed out that patients’ migration background could differentially influence these needs (quote #05).

***#04*** *“And, it’s a clincher, but every patient is unique. .. of course that doesn’t mean that you cannot define certain groups with shared characteristics or needs. But ideally you want to give care in such a way that it is appropriate to that patient. And that fits very well with palliative care, in which you want care to be appropriate to what matters to that patient, what is quality of life for that patient.” (Researcher 2018.12)*

***#05*** *“So maybe we shouldn’t be fixated on that, but instead say responsive to diversity. That does of course sounds like an umbrella term, so we’d have to call it something else. But if you’re talking about patient-centred care then you have to look at someone’s background. What matters to someone … and your country of origin does affect that I think” (Researcher 2018.10)*

Many researchers saw their projects as a way to support professionals in the provision of patient centred care and would encourage them to consider patients’ migration background (quote #06). However, some researchers realized that limited awareness of factors that cause patients with a migration background to be underserved might cause a bias (quote #07). E.g. religion is included as a research variable because this is a factor *known* to play a role in palliative care. Other factors may remain unaddressed. Researchers did mention they expect sociodemographic changes such as the aging of migrant populations to increase the perceived need for responsiveness efforts from the start of their projects.

***#06*** *“And especially in palliative care you have to consider the person, patient-centred care. So theoretically speaking you would naturally consider someone’s background. I also know of course that this is the case only ‘theoretically speaking’. If we don’t provide healthcare professionals with extra knowledge, or pay attention to these groups it will get lost in … and will never land on the level of the healthcare professional.” (Researcher 2017.09)*

***#07*** *“Yes, I think ideally as a researcher you portray a reflection of reality. That’s my ideal too. But at the same time we know that as a researcher you are limited by our own bias and sometimes also by the limited knowledge you have of the study population.” (Researcher 2018.10)*

Another way in which some researchers understood responsiveness is from an equity perspective. These researchers reasoned underserved groups, in general, require special attention in their projects because ‘they would benefit the most’. These researchers intentionally aimed to improve palliative care for underserved groups (quote #08).

***#08*** *“See, our project was set up deliberately for older adults because we had the feeling that the younger patient groups, the oncology patients, are attended to. It’s the older adults that are forgotten. And to deliberately consider which groups should be attended, but aren’t. Or, indeed, to consider which patients with certain conditions you have sitting in your waiting room, but you find it difficult to address the topic … that would be a good start, to tend to those.” (Researcher 2017.13)*

#### Experience

Researchers’ experience with responsiveness measures varied. A more limited experience in conducting responsive research affected their ability to contribute to responsiveness of palliative care through their projects in several ways: the measures researchers had taken did not target underrepresented populations, e.g. limited experience with patient participation lead to participation of patients belonging to groups that were already well represented; assumptions and scepticism researchers held about responsiveness measures for patients with a migration background had not been challenged (quote #09); and, researchers experienced uncertainty with regards to what actions to take, what options they had, etc. (quote #10). Conversely, researchers who were more experienced listed more options of actions they could take to improve responsiveness in their projects.

***#09*** *“Until now we have very often not included it as a research question because you’d have to do so many extra things. So often we do include it as a variable, but then we also see that actually we don’t … or that we only include a few people.” (Researcher 2017.12)*

***#10*** *“And in some way you also want to be inclusive of course. But maybe we are also lacking a clear picture, or the knowledge and experience of how to tailor communication in healthcare, and specifically in the palliative phase too, with the different types of patients. The highly educated, the Dutch speaking, the patients with low health literacy and the non-western migrants. How … If you want to tend to all their communication needs, what does our tool need to be? I, for example, think it needs to be much more visual. I am not sure, but this is what I come up with. Much less text, much more visual. And the remaining text is legible for low-literate people, so a little bit … [layman’s terms], short sentences. But if that also tends to the needs of non-western migrants? Well I don’t know.” (Researcher 2018.15)*

Another way in which experience, or lack thereof, influenced researchers’ efforts in contributing to palliative care responsive to patients with a migration background is reflected in quote #11. It explains that, while working to innovate palliative care, researchers look for a starting point that has the highest likelihood for the innovation to be successful. It may have been a conscious choice to defer responsiveness of research to such underrepresented groups to a later stage. Oftentimes, however, it was an automatism.

***#11*** *“I think the approach is quite unique and it can’t hurt to consider that for a moment. I think sometimes it’s also almost an automatism to first try out with patients with whom you think it will land well. And those, again, are the highly educated patients, white people. And I do indeed see a responsibility there to consider that ‘these are not the only people we serve’.” (Researcher 2017.13)*

### Institutional level

Besides factors on the individual level, researchers discussed several factors outside their sphere of influence. Possibilities to contribute to responsiveness in the research project depended on the project’s scope; projects were limited by time, money and the number of locations where care innovations were implemented (quote #12). Researchers furthermore mentioned their ability to contribute to responsiveness to patients with a migration background was influenced by the interaction with healthcare professionals, healthcare institutions, research institutions and funding agencies.

***#12*** *“A lot of questionnaires need to be filled out per patient, there are about three questionnaires for the patient and one for their informal caretaker, and they all need to be filled out every month. They are filled out by the nurse that does the visits on behalf of the hospital and it is impossible for them to do this in another language or to bring someone along to all these visits. Those are very big expenditures which we did not include in our budget. So that’s a pity.” (Researcher 2017.11)*

#### Interaction with healthcare professionals and healthcare institutions

Researchers indicated that, at times, creating palliative care responsive for patients with a migration background in their projects was not entirely in their control. They dealt with healthcare institutions that may not have certain policies in place, e.g. interpreter services. And some of the care innovations were simultaneously implemented through initiatives outside their project. Researchers sought collaboration, or used materials from those initiatives. However, these initiatives may not prioritize efforts to improve responsiveness for patients with a migration background.

On the other hand, researchers sometimes held assumptions about the responsiveness of others, e.g. healthcare professionals involved in their project (quote #13). This affected researchers’ readiness to take their own measures as well as raise the subject among other actors.

***#13*** *“I think it also has to do with the fact that I assume general practitioners, in their training, have … or if it’s a GP who works in a culturally diverse neighbourhood … that that person has acquired skills by other means. That our project won’t be the first time they are confronted with diversity.” (Researcher 2017.13)*

Collaboration with healthcare professionals at times gave researchers input on the responsiveness of the palliative care innovations (quote #03). Furthermore, the collaboration with healthcare professionals was vital for the type of projects that the researchers were involved in; healthcare professionals often played an important part in enrolling patients in a project. This created a precarious relationship which influenced researchers’ efforts to improve responsiveness for patients with a migration background in various ways.

Healthcare professionals’ contribution to enrolment of patients often required them to introduce the topic of palliative care to patients. Researchers explained some healthcare professionals may not recognize the palliative phase and omit to enrol patients for that reason. Or, if they do recognize the palliative phase, having to conduct a conversation on palliative care with patients with a migration background may be a barrier as it potentially affects their care relationship (quote #14). Furthermore, having to introduce a research project following such a conversation could be seen by the healthcare professional as additionally burdening the patient. These deliberations finally prompted researchers to express that requiring healthcare professionals to do all this in a ‘culturally sensitive’ manner was a bridge too far.

***#14*** *“I think the risk is that, indeed, you are not even going to introduce it to patients with a non-western migration background if you are going to experience negative consequences as a healthcare professional. Because I do hear from healthcare professionals that they have negative experiences with that, that they jeopardize their care relationship. And then you practically impose, you will assess, am I going to impose something or am I going to introduce something that I, because of my western background, think is a good initiative, but may jeopardize my care relationship? Or will I not do it?” (Researcher 2018.11)*

Researchers wanted to facilitate healthcare professionals as much as possible and were hesitant to request any efforts to improve responsiveness that would cost extra time (quote #15). Even if researchers found it important to raise awareness of lack of responsiveness to patients with a migration background amongst healthcare professionals, they were frugal in their efforts (quote #16).

***#15*** *“I think it is the responsibility of the institutions professionals work for, strictly speaking. But that doesn’t mean that we can’t encourage something. At the same time we have to be careful that we don’t overwhelm them (the professionals) with things which we think they should do.” (Researcher 2017.09).*

***#16*** *Well, I think it very much depends on which healthcare professionals you expect to work in a culture sensitive manner. I think it will be very hard to expect this of medical specialist and specialists in training in a hospital. I think you can ask the palliative consultation team because the principles of palliative care – medical, psychological, existential and spiritual – allow it. Within these principles it is very important to discuss what the patient wants, and what the patients wants is very much dependent on their culture and religion. So that is very much related. I don’t see a lot of resistance against including it there.” (Researcher 2017.11)*

#### Interaction with research institutions and funding agencies

Researchers’ efforts to improve responsiveness to patients with a migration background were furthermore influenced by the interaction with research institutions. Scientific research needs to adhere to certain ‘scientific standards’. Institutions such as medical ethical committees and funding agencies monitor adherence to these standards. They thereby establish what are accepted practices in scientific research. One example given by a researcher involved in an RCT was that they were not able to purposively (over) sample specific groups; they were obliged to “*include everyone we encounter. As many as we can. And you cannot say we’ll skip that Dutch patient because … You want everyone.”* In quote #17 the researcher explained that scientific standards i.e. written informed consent, in Dutch, create a barrier for responsiveness to patients with a migration background.

***#17*** *“And at the same time I also do realize it is not always quite possible to take diversity into account. Especially when you do things like an RCT, in which you know ‘this is what I have to do on paper’ and ‘it has to be in Dutch’ or what … those kinds of things.” (Researcher 2017.09)*

Conversely, researchers expressed, institutions such as funding agencies could positively reinforce their contribution to responsive palliative care by emphasizing its importance, setting responsiveness requirements for project proposals or issuing specific calls for research involving underrepresented populations (quote #18).

***#18*** *“But it has to be fair. If in the end we look at the entire budget plan and the expenditures are way too high partially due to interpreter services of which I think … ‘This is not my main research question.’ then chances are quite high that that is one of the expenses we immediately cut again. Unless, and that is something the funding agency does more and more, you are reminded of how important it is.” (Researcher 2017.12)*

The project this study was part of exemplifies how emphasizing the importance of responsiveness helps researchers in addressing it. The project was funded by the national program for palliative care innovation, like the projects by researchers in our study, and aimed to help researchers assess and find ways to improve their projects’ responsiveness to diversity, and specifically to patients with a migration background. Researchers expressed that our project helped them get responsiveness on the agenda and take action to improve it (quote #19). MT had extensive knowledge about responsiveness measures and researchers indicated that the very practical support, or information on where to get that support, helped to take action.

***#19*** *“But the fact that you come by serves as a big stick. That I could consult you about the letter and that later on we can, hopefully, ask you questions about the training; if what we want to do is good or not. .. The fact that we have such a source and stick makes that it gets done and that you … You never get that awareness if it is limited to a small reminder every half year that goes ‘oh right, we should have included this, but now it’s already too late.’.” (Researcher 2018.13)*

Researchers listed downsides to the current structures put in place by funding agencies to ensure responsiveness to patient diversity. These could result in researchers merely paying lip service; including diversity in project proposals without making sustainable improvements to responsiveness; and, researchers expressed the role of the funding agency was at times equivocal (quote #20).

***#20*** *“But sometimes you can do it, especially if it is a large project. That you come up with something and start a sub project to research the effectiveness amongst other groups. But it has a downside. We have previously done it in project proposals, which in fact didn’t make it. That it was possible within the budget, and then I also think it’s very interesting to do. But those proposals didn’t make it and I am not sure … It could also be that they don’t make it because it makes the proposal less straight forward. And that the funding agency prefers a straight forward proposal. I am not sure if that was the case for this particular project, but I do suspect it. And then it almost works against you that you take into consideration who you don’t include. I often think you have to keep it as simple as possible, otherwise it does not work to your advantage.” (Researcher2017.09)*

## Discussion

Our study shows that researchers’ efforts to address responsiveness of palliative care to patients with a migration background and other underserved populations in their projects is subject to factors at the individual level, i.e. researchers’ awareness, perception and experience as well as at the institutional level, i.e. in the interaction with healthcare institutions, healthcare professionals, research institutions and funding agencies. These resemble factors prevalent in other types of research [12, 22], but at times play out differently in their influence on palliative care research.

On the individual level, researchers’ limited awareness of the need for responsiveness to patients with a migration background, especially in the context of palliative care innovations, limits their efforts to be responsive. This ‘unconscious incompetence’ becomes ‘conscious incompetence’ when researchers’ awareness increases, but researchers’ limited experience can continue to prevent them from engaging in responsiveness efforts [23]. The idea of a competence cycle stems from learning theory; it posits learners enter the phase of ‘conscious incompetence’ when they start to get a sense of their own limitations; upon realization can slowly begin to acquire ‘conscious competence’; and in time mastery will become automatic and they work mainly through intuition and past experience – ‘unconscious competence’ [23, 24]. Our findings suggest it is more commonplace for researchers in palliative care to work on responsiveness to groups more traditionally known as underserved in palliative care then to focus on patients with a migration background. Traditionally, palliative care has been modelled on the relatively younger cancer patient [25]. The researchers we interviewed made a conscious effort in their projects to increase the reach of palliative care to include patients with a non-cancer diagnosis, or older adults. With regards to these groups, researchers had reached the stage of conscious competence.

Our findings demonstrate that researchers differ in how they understand responsiveness and the efforts it requires. Some of the researchers in our study equated responsive care to patient centred care – a strategy to improve patients’ experiences of the way health care is delivered in the interaction between practitioner and patient [26]. In their view the contribution of research to responsive palliative care is to provide an evidence base which informs professional practice and enables practitioners to deliver patient centred care. However, their research (the choice of interventions, research questions, etc.) and thus the knowledge they generate is driven – and partially limited – by their awareness of underserved groups in the study population. This limitation can further be illustrated by the notion of research as a *situated cultural practice* [27]. The researcher as an individual and a member of a scientific field has a sociocultural location which mediates how they think, ask questions, collect and interpret evidence, and report findings.

Furthermore, the accepted practices - “actions that are repeated, shared with others in a social group, and invested with normative expectations and with meanings or significances that go beyond the immediate goals of the action” - of the scientific field are based on cultural presuppositions [27]. Our findings show that researchers at times felt limited in their efforts to improve responsiveness for patients with a migration background by certain scientific standards they have to adhere to. These standards are reinforced by medical ethical committees and funding agencies and become established as accepted practices in scientific research. Flexibility by researchers and research institutions to alter habitual practices – to acknowledge that community involvement and partnership, extended timeframes, higher resourcing costs, multiple recruitment strategies, flexibility in data collection, dissemination of research findings using accessible language and focusing on practical implications, etc. are required – is necessary for responsiveness of palliative care research [12, 15, 19].

Our study thus shows that, on the institutional level, research institutions can constrain responsiveness efforts. It also suggests funding agencies can encourage responsiveness to patients with a migration background by setting responsiveness requirements. Previous research has shown that researchers who received funding from sources with minority participant inclusion and reporting mandates indeed used more diverse and active recruitment strategies [22].

Finally, our study shows that efforts of researchers to improve responsiveness of palliative care to patients with a migration background occur alongside and are contingent upon efforts of healthcare institutions and healthcare professionals. Ultimately, promoting responsiveness to diversity requires interventions at several levels of the health system [17]. Researchers are dependent on whether healthcare organisations have diversity elements in policies and management in place, and on healthcare professionals’ ability to enrol patients. Our findings confirm previous findings and suggest that in palliative care research in particular enrolment of patients with a migration background is compounded by healthcare professionals’ inability to identify the palliative patient and address the topic of palliative care [11].

### Limitations

Our findings are based on interviews with researchers and represent their perspective on factors influencing their efforts to improve responsiveness. Our findings are therefore limited to the factors researchers were aware of; there may be additional unreported factors that researchers are unaware of [24]. Furthermore, this study was part of a larger project to assess and find ways to improve responsiveness of palliative care research to patients with a migration background [20]. Researchers’ participation in this project increased their awareness of and encouraged them to take action on responsiveness, this may have impacted some of our findings. Lastly, our study was conducted in the context of the Netherlands and the national program for palliative care innovation (Palliantie. Meer dan Zorg). Responsiveness of palliative care services and research in other countries may be subject to different influencing factors.

## Conclusion and implications

Researchers play a key role in ensuring research demonstrates responsiveness to patients with a migration background and other underserved populations in order to obtain representative research findings and allow the development of an evidence base that can be used by service providers and policy makers to reduce disparities in palliative care. Their efforts are affected by the interaction with research institutions, healthcare institutions and healthcare professionals. Our research suggests there are several ways to increase opportunities to improve responsiveness of palliative care through research. To address individual level factors we recommend training in responsiveness for researchers in the field of palliative care; to increase knowledge of patients with a migration background and other underserved populations in palliative care and familiarity with responsiveness measures. To address factors on the institutional level we also recommend training for healthcare professionals involved in palliative care research projects; to learn to address the topic of palliative care and increase enrolment of patients with a migration background and other underrepresented populations. Lastly, we encourage researchers as well as research institutions and funding agencies to allow flexibility in research practices and set a standard for responsive research practice. Providing the opportunity for practical support helps researchers to get responsiveness of palliative care on the agenda and take action to address it.

When such opportunities to improve responsiveness of research are utilized, research can help identify and understand determinants of disparities, identify and evaluate interventions to eliminate them and contribute to quality improvement and innovation of equitable palliative care, in which patients and families receive care according to their needs.

## Supplementary Information

**Additional file 1.** Interview guide. Semi-structured interview guide used for interviews. The interview guide includes questions on researchers’ perception of diversity, responsiveness and their role in contributing to responsiveness of palliative care in their projects. It also includes suggestions for strategies to improve responsiveness roughly following the domains of the equity standards – equity in policy; equitable access and utilisation; equitable quality of care; equity in participation; and, promoting equity – used to discuss the feasibility of implementing such strategies and identify factors influencing researchers’ effort to contribute to responsiveness of palliative care in their projects.

## Data Availability

The datasets generated and analysed during this study are available from the corresponding author on reasonable request.
