# Supplementary material for: The role of research in improving responsiveness of palliative care to migrants and other underserved populations in the Netherlands: a qualitative interview study
Source: BMC Palliat Care. 2021 Jan 6;20:5. doi: 10.1186/s12904-020-00701-2 (PMC7789154; doi:10.1186/s12904-020-00701-2)
Supplement: Supplementary file 1 — Additional file 1. Interview guide. Semi-structured interview guide used for interviews. The interview guide includes questions on researchers’ perception of diversity, responsiveness and their role in contributing to responsiveness of palliative care in their projects. It also includes suggestions for strategies to improve responsiveness roughly following the domains of the equity standards – equity in policy; equitable access and utilisation; equitable quality of care; equity in participation; and, promoting equity – used to discuss the feasibility of implementing such strategies and identify factors influencing researchers’ effort to contribute to responsiveness of palliative care in their projects. [file 12904_2020_701_MOESM1_ESM.pdf]

The role of research in improving responsiveness of palliative care to migrants and other underserved populations: a qualitative interview study

INTERVIEW GUIDE

[General]

1. Can you briefly tell me about the palliative care research you are working on?

2. Why did you decide to participate in our project (a project to help researchers assess and find ways to improve their projects' responsiveness to diversity, in light of the aging migrant population)?

3. How would you define diversity? What is, in your view, the extent of diversity that you can reasonably take into account in your research?

Working definition of diversity

"From here on onwards, when we speak about diversity, we are referring to (older) adults with a non-western migration background. They are a diverse group with regards to ethnicity, culture, religion, educational level and literacy level. This diversity can be extended further, especially if we look beyond the first generation. To be able to respond to a social issue such as the ethnic disparities in palliative care, however, we will focus on the population groups that currently suffer the consequences of these ethnic disparities which leads us to predominantly focus on older migrants of the first generation with a non-western background, i.e. Surinamese, Turkish or Moroccan background."

[Design]

4. Did the diversity of the patient population come up when designing your research?

5. Which steps did you take to ensure responsiveness to diversity in your research?

> *What were reasons to take these steps? What were reasons to not take steps?*
>
> *Did you experience barriers in taking these steps?*

6. Are there external factors that influence your efforts to ensure responsiveness to diversity in your research?

7. As a researcher, what do you feel is your responsibility in improving diversity responsiveness of palliative care?

Can you do enough? Is it an option not to do anything?

How will you know if the palliative care innovation resulting from your research is responsive to the needs of patients with a non-western migration background?

[Execution]

8. Which barriers to diversity responsiveness do you come across while doing your research?

When you don't come across barriers, does that mean there are no barriers or does it mean that you do not come across (reach) diverse patients?

9. How can diversity responsiveness be improved?

Looking back and looking ahead?

Looking at the domains of the equity standards (see textbox)?

What are quick wins? What are structural improvements?

10. How can you, as a researcher, and the people and institutions involved in performing your research be facilitated in improving diversity responsiveness?

How do you, as a researcher, relate to the people and institutions involved in performing your research? How does the collaboration with palliative care providers work?

Who gives instructions for improving responsiveness?

11. Is this (research as an entryway) a good way to sustainably achieve improving diversity responsiveness of palliative care?

Working definition of diversity

“Let’s extend our definition of diversity further, and look beyond the first generation. The term super-diversity has been coined to describe changing migration patterns and their impact on society. On the one hand there is an increase in the extent of diversity, following migration from an increasing number of different countries. On the other hand, there is a growing diversity within this diversity. A multitude of countries of origin, languages, cultures, religions and social statures creates diversity between and within groups and societies. The complexity of and interaction between these factors is the core of the term super-diversity.

Following this line of reasoning responsiveness to (super) diversity means looking beyond single target groups (e.g. non-western migrants) and taking into account the interaction between all factors that contribute to disparities.”

12. How would you deal with responsiveness to diversity in this light?

13. What barriers and facilitators can you thing of?

Textbox: Domains of the equity standards

Standard 1: Equity in Policy (Policy of the research project)

This domain pertains to the choices with regards to diversity responsiveness made in the set-up of the project (e.g. choice of inclusion criteria, choice of budget spending), the composition of the research project, and the monitoring of access to and quality of care for a diverse patient population.

Standard 2: Equitable Access and Utilisation (Access to the research project)

This domain pertains to the opportunity to participate in the research project. Is a diverse patient population informed about opportunities to participate proactively, and with understandable information? Are questionnaires and other measurements validated and available in other languages? This domain also pertains to geographical accessibility.

Standard 3: Equitable Quality of Care (Quality of care within the research project)

This domain pertains to the quality of palliative care offered as part of the research project. Are palliative care professionals culturally competent, are language barriers overcome, e.g. by using interpreters?

Standard 4: Equity in Participation

This domain pertains to the involvement of patients in the various stages of a research project. Is there a patient panel, has an effort been made to ensure this is a diverse panel? Is the panel consulted in various stages of the research project?

Standard 5: Promoting Equity

This domain pertains to the collaboration with other parties in improving diversity responsiveness of palliative care. Are experiences with regards to improving diversity responsiveness in the research project actively shared?
